# The impact of obesity on sleep, pulmonary and chest wall restriction in Osteogenesis Imperfecta: a pilot study

**DOI:** 10.1186/s13023-024-03489-z

**Published:** 2024-12-20

**Authors:** Ramona De Amicis, Vittorio Landoni, Simona Bertoli, Alessandro Sartorio, Andrea Aliverti, Antonella LoMauro

**Affiliations:** 1 Department of Food, Environmental and Nutritional Sciences (DeFENS), International Center for the Assessment of Nutritional Status and the Development of Dietary Intervention Strategies (ICANS-DIS), University of Milan, 20133 Milan, Italy; 2 Obesity Unit and Laboratory of Nutrition and Obesity Research, Department of Endocrine and Metabolic Diseases, IRCCS Istituto Auxologico Italiano, 20145 Milan, Italy; 3 Valduce Hospital - Villa Beretta Rehabilitation Center, 23845 Costa Masnaga, Lecco Italy; 4 Experimental Laboratory for Auxo-Endocrinological Research, IRCCS Istituto Auxologico Italiano, 28824 Piancavallo - Verbania, Italy; 5 Dipartimento di Elettronica, Informazione e Bioingegneria, Politecnico di Milano, Piazza Leonardo da Vinci, 20133 Milan, Italy

**Keywords:** Osteogenesis Imperfecta, Spirometry, Opto-electronic plethysmography, Obstructive sleep apnoea syndrome, Fat mass, Body composition, Obesity

## Abstract

**Introduction:**

Osteogenesis Imperfecta (OI) is characterised by brittle bones, severe skeletal deformities, low sleep quality, and restricted breathing. We aimed to distinguish how disease and obesity affect these results.

**Methods:**

According to BMI, we considered four groups of peer adults (median age: 35.0 years): 13 subjects affected by moderate or severe OI without obesity (OIno), 14 affected by moderate or severe OI with obesity (OIob), 10 without obesity not affected by OI (OB) and 10 without obesity not affected by OI.

**Results:**

Obstructive Sleep Apnoea Syndrome was diagnosed in 4 OIno (30%) and 9 OIob (64%). Restricted lung pattern (z-score of total lung capacity < − 1.64) was diagnosed in 10 OIno (77%); 9 OIob (65%), and 1 OB (10%) subjects. In the seated position, OIob breathed with reduced tidal volume and higher respiratory rate, resulting in hypoventilation. Both OIno and OIob were characterised by rapid and shallow breathing and lower ribcage expansion (negative in 3 (23%) OIno and 3 (21%) OIob). In the supine position, the ventilatory pattern was similar among the four groups, while both OIno and OIob were characterised by reduced ribcage contribution, which was negative in 6 (46%) OIno, 11 (78%) OIob and 1 (10%) OB.

**Conclusions:**

This is a pilot study on a small sample, the findings and conclusions apply only to this study population. The preliminary results suggest that in subjects with moderate or severe OI per se implies (1) a 30% prevalence of obstructive sleep apnoea syndrome, (2) a restricted lung pattern, (3) a lower ribcage expansion, and (4) rapid and shallow breathing in the seated position. The additional impacts of obesity on OI seem to determine (1) a higher incidence of obstructive sleep apnoea syndrome, (2) hypoventilation in the seated position, and (3) a higher incidence of paradoxical breathing lying supine. Reversing obesity in OI is even more challenging as knowledge of the diet and the physical activity suited for these patients is still scarce.

**Supplementary Information:**

The online version contains supplementary material available at 10.1186/s13023-024-03489-z.

## Background

Osteogenesis Imperfecta is a rare disorder occurring in 1 in 15 k to 20 k births [1] that affects the connective tissue causing extremely fragile and deformed bones [2]. According to clinical and radiological evaluation, the severity of Osteogenesis Imperfecta is grouped into four subtypes: type III represents the most non-lethal form, while type IV and I are the moderate and mild forms, respectively [3]. Independently on the form, respiratory failure is the most common cause of death in Osteogenesis Imperfecta [4, 5]. Osteogenesis Imperfecta is classified as a restrictive lung disease characterized by reduced lung volumes [6–16]. In Osteogenesis Imperfecta, the restriction is mainly due to problems related to the expansion of the chest wall during inhalation, while the reduction in lung elasticity is still to be confirmed [17]. Respiratory restriction is often aggravated by obesity. A systematic chest wall restriction in the supine position was recently found to characterise adults with essential obesity, mainly due to reduced ribcage expansion [18]. Ribcage restriction is also typical in Osteogenesis Imperfecta, starting in childhood [10] and evolving during adulthood to the point of determining paradoxical inspiratory movement in type III form [10, 11, 15]. The intrinsic factors predisposing subjects with Osteogenesis Imperfecta to restricted ribcage are structural modifications of the spine and the ribcage, namely pectus carinatum [10, 16]. In addition, reduced physical activity combined with abnormalities in nutritional status predispose these patients to excess weight and related problems [19]. We have recently shown that the pathophysiology of Osteogenesis Imperfecta creates a dangerous vicious circle among breathing, sleep, and nutritional status, leading to a high incidence of obstructive sleep apnoea associated with obesity and paradoxical breathing [15]. However, we could not separate the two potential factors (i.e., disease and obesity) affecting those results. This pilot study aims to evaluate lung function, breathing pattern, and quality of sleep by considering four groups of subjects, with and without obesity, with and without Osteogenesis Imperfecta, to try to understand the impact of obesity on these functions. The rationale of this new study was that both Osteogenesis Imperfecta and obesity are associated with obstructive sleep apnoea and thoracic restriction. Sleep disturbance seems to be an underdiagnosed symptom in the population of Osteogenesis Imperfecta [15, 20]. Paradoxical thoracic breathing in the supine position can further aggravate sleep quality. For these reasons, it is crucial to distinguish the role of the disease and obesity on respiratory function and sleep for better management of individuals with Osteogenesis Imperfecta.

## Methods

### Population

Patients with Osteogenesis Imperfecta were enrolled on a voluntary basis by As.It.O.I., the Italian Association of Osteogenesis Imperfecta. Inclusion criteria were a confirmed Osteogenesis Imperfecta type III (severe) and IV (moderate) diagnosis, stable condition, absence of cardio-respiratory pathologies and willingness to participate in the study.

Subjects with essential obesity were recruited at the Division of Metabolic Diseases, Istituto Auxologico Italiano, IRCCS, Piancavallo-Verbania, Italy, where they were hospitalized for a multidisciplinary body weight reduction program.

Healthy subjects without obesity were recruited among colleagues and friends of the authors. Inclusion criteria were the absence of cardio-respiratory pathologies and willingness to participate in the study.

Informed consent was obtained from all the subjects. The study was conducted according to the statement of the Declaration of Helsinki. The protocol and the data analysis were approved by the Ethical Board Committee of Politecnico di Milano, Italy (protocol number: 47/2021) for Osteogenesis Imperfecta patients and normal weight subjects and by the Ethical Committee of Istituto Auxologico Italiano, Milan, Italy (research code: 01C307-2013; acronym: POSTVOLOB) for the subjects with essential obesity.

### Body composition

Body weight (BW, kg) and body height (BH, cm) were measured with 100 g and 0.5 cm accuracy, respectively. Body mass index (BMI) was calculated using the formula BW (kg)/BH^2^ (m^2^). Normal weight was defined as a BMI between 18.5 and 25 kg/m^2^ [21]. Waist circumference was measured in the supine position at the end of a normal exhalation with an inextensible tape, with millimetre precision. The measurement was taken in a horizontal plane at the midpoint between the last rib and the iliac crest [22].

All patients with Osteogenesis Imperfecta and subjects with essential obesity underwent dual-energy X-ray absorptiometry (DEXA) scans (iDXA; General Electric, formerly Lunar Corp., Madison, WI) to measure bone mineral content (BMC) and soft tissue, including fat mass (FM) and lean body mass (LM).

The ratio between total body fat mass and total body mass (fat + lean mass + bone mass of total body) multiplied by 100 was the fat mass percentage (FM%); while the fat-free mass (FFM) was calculated by adding BMC to LM. Certified research staff performed the DEXA scans with patients lying supine on the table, their feet in a neutral position, and arms resting along their sides. The radiation exposure was < 7 mSv for an average measuring time of 10 min. The DEXA scans of Osteogenesis Imperfecta patients were analysed using custom-made software that allows body composition measurements in close relation to metal orthopaedic implants (typical in Osteogenesis Imperfecta) by excluding non-osseous pixels.

### Lung volumes and nocturnal oxygen saturation

Forced vital capacity (FVC) was measured through spirometry (Vmax series 22, SensorMedics, Yorba Linda, CA). Total lung capacity (TLC) was measured through the nitrogen washout technique (Vmax series 22, SensorMedics, Yorba Linda, CA). The predicted values were computed following the Global Lung Initiative [23].

According to the ERS criteria, lung restriction was identified when the percentage value of TLC was below the 5th percentile (corresponding to 1.64 relative standard deviation) [24].

Nocturnal oxygen saturation was measured using a digital pulse oximeter (Nonin, 8500 digital pulse oximeter Quitman, TX) to diagnose obstructive sleep apnea syndrome (OSAS). OSAS was present when the AHI index (i.e.: the number of apnea and hypopnea events per hour of sleep) was ≥ 5 [25].

### Ventilatory and thoraco-abdominal pattern at rest

**Fig. 1:**
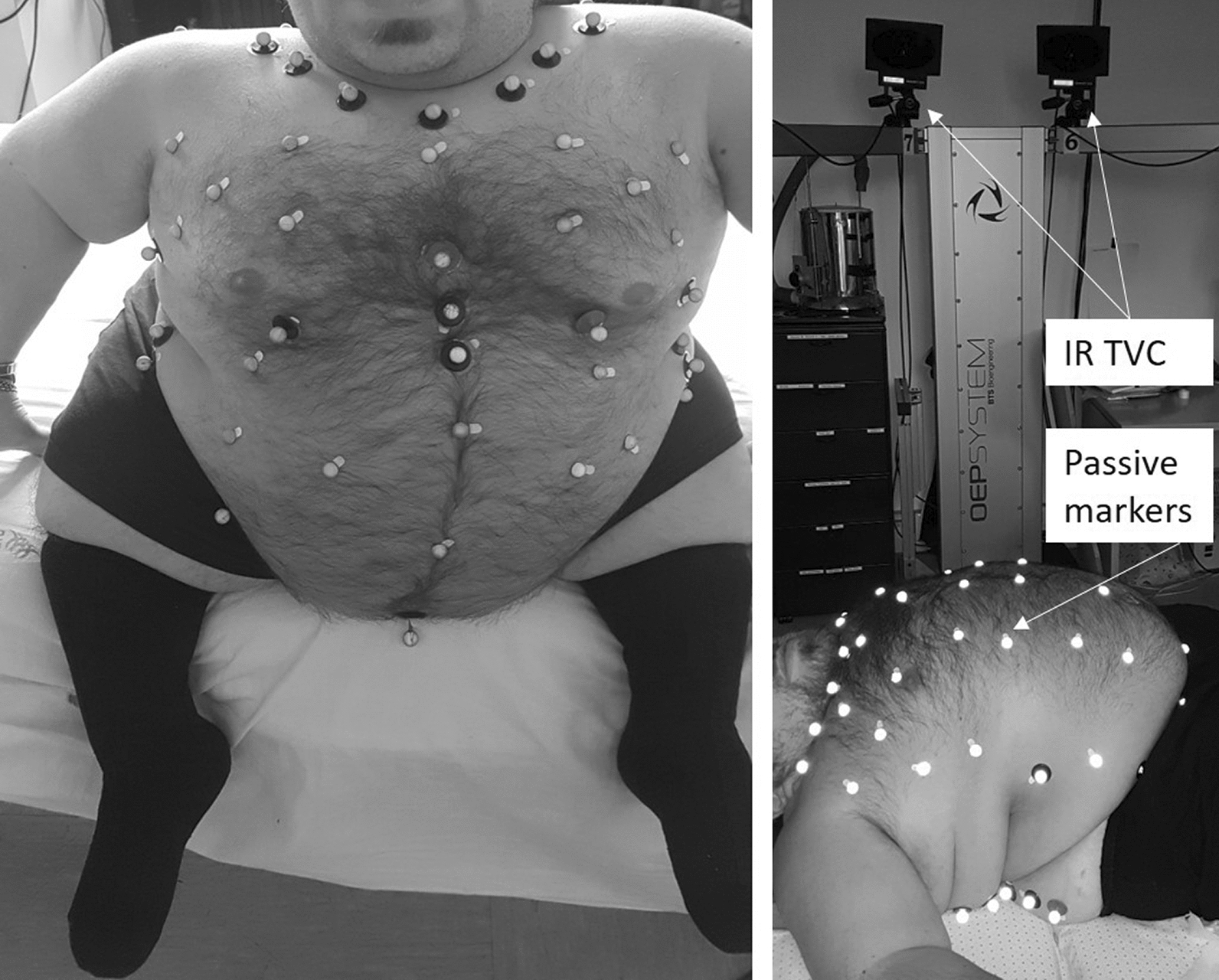
Exemplificative photo of the opto-electronic plethysmography set-up for assessing breathing patterns in seated (left panel) and supine (right panel) positions. Photos were published with the permission of the patient

Opto-electronic plethysmography accurately assessed the ventilatory and thoraco-abdominal pattern during spontaneous awake quiet breathing (OEP System; BTS, Milan, Italy) in the seated and supine positions (Fig. 1).

At least one minute of stable spontaneous breathing was identified and an average breath was computed. The following parameters were computed on the average breath: tidal volume normalized according to the body weight, respiratory rate, minute ventilation (as the product of the two), rapid and shallow breathing index (as the ratio of the two), and the percentage ribcage contribution to tidal volume.

### Statistical analysis

The Kolmogorov–Smirnov test was used to verify the data distribution. When both the normality and equal variance tests passed, the One Way Analysis of Variance was used with the four groups of subjects as independent variables. All pairwise multiple comparison procedures implemented the Holm-Sidak method.

When the normality or equal variance test failed, the Kruskal–Wallis One Way Analysis of Variance on Ranks was used with the four groups of subjects as independent variables. All pairwise, multiple comparison procedures implemented the Dunn's Method (SigmaStat version 11.0; Systat Software, San Jose, Calif., USA).

Significance was set as *p* < 0.05. Data in the results section of the text are reported as median values (25th and 75th percentiles).

## Results

### Subjects

Forty-seven subjects were recruited for this study (age: 35.0 (30.3–46.5) years; height: 1.45 (1.2–1.7) m; weight: 65.0 (44.7–85.0) kg; BMI: 30.9 (24.2–39.3) kg/m^2^), 27 of whom had a confirmed diagnosis of moderate or severe Osteogenesis Imperfecta.

According to BMI, four groups were identified: 13 subjects without obesity affected by moderate or severe Osteogenesis Imperfecta (OIno; 8 women; 7 type III; 9 wheelchair-bound patients), 14 subjects with obesity affected by moderate or severe Osteogenesis Imperfecta (OIob; 10 women; 7 type III; 11 ambulant patients), 10 subjects with obesity not affected by Osteogenesis Imperfecta (OB; 5 women) and 10 subjects without obesity not affected by Osteogenesis Imperfecta (NO; 4 women). The four groups were similar in age (Table 1).

**Table 1:** Anthropometric data, body composition status, breathing rate and tidal volume in patients without obesity affected by Osteogenesis Imperfecta (OIno), in patients with obesity affected by Osteogenesis Imperfecta (OIob), in subjects with essential obesity not affected by Osteogenesis Imperfecta (OB) and in otherwise healthy subjects without obesity not affected by Osteogenesis Imperfecta (NO)

	OIno				OIob				OB				NO		
	Median	25p	75p		Median	25p	75p		Median	25p	75p		Median	25p	75p
Age (yrs)	32.1	17.2	44.7		40.1	33.6	44.5		38.0	34.0	46.3		37.5	25.3	50.0
Weight (Kg)	40.0	26.6	50.4	* vs OB,NO	57.0	43.2	66.1	* vs OB	112.9	100.3	138.3	* vs all	71.0	61.8	75.8
Height (m)	1.26	1.04	1.43	* vs OB,NO	1.24	1.01	1.34	* vs OB,NO	1.67	1.56	1.76	* vs Olno,Olob	1.74	1.70	1.78
BMI (Kg/m^2^)	24.8	24.0	26.5	* vs OB, Olob	35.7	32.2	41.2	* vs Olno,NO	40.5	38.7	48.0	* vs Olno,NO	22.9	20.9	24.1
Waist circumference (cm)	71.5	70.0	78.7	* vs Olob,NO	103.1	83.1	111.7	* vs all	132.0	118.8	143.0	* vs all	83.3	74.9	86.1
Waist/Height	0.64	0.50	0.70	* vs all	0.78	0.74	0.86	* vs Olno,NO	0.73	0.71	0.90	* vs Olno, NO	0.47	0.43	0.49
FM (%)	34.00	26.75	36.98	* vs Olob,OB	47.30	41.50	51.90	* vs Olno	48.35	40.73	50.90		–		
FFM (%)	65.08	61.96	72.34	* vs Olob,OB	51.49	46.76	57.55	* vs Olno	51.65	49.10	59.28		–		
FM /FFM	0.54	0.38	0.61	* vs Olob,OB	0.94	0.74	1.14	* vs Olno	0.94	0.69	1.04		–		
*Seated position*															
Breathing rate (breaths/min)	17.88	3.55	2.78		23.25	4.96	3.51	* vs Olno, NO	17.91	2.54	4.31		18.09	3.65	2.36
Tidal volume (mL/Kg)	0.71	0.21	0.12		0.35	0.09	0.13	* vs Olno,NO	0.53	0.04	0.09		0.92	0.17	0.18
*Supine position*															
Breathing rate (breaths/min)	18.37	1.95	3.53		21.76	6.34	2.62		16.86	2.95	5.99		15.57	4.34	3.63
Tidal volume (mL/Kg)	0.72	0.15	0.28		0.53	0.17	0.24		0.50	0.05	0.10		0.61	0.03	0.13

BMI, body mass index; FM, fat mass; FFM, fat free mass; 25p, 25th percentile; 75p, 75th percentile; * *p* < 0.05

### Body composition

OB was the heaviest group. Height was lower in both groups of patients affected by Osteogenesis Imperfecta. Waist circumference, the percentage of fat mass, and its ratio with the fat-free mass were higher in both groups with obesity compared to those without obesity. There were no differences in terms of BMI and body composition between OB and OIob.

By definition, the BMI of the two groups of individuals with obesity was higher than that of the two groups without obesity. The BMI was similar between the two groups of subjects with obesity and between the two groups of subjects without obesity (Table 1).

### Lung volumes and nocturnal oxygen saturation

**Fig. 2:**
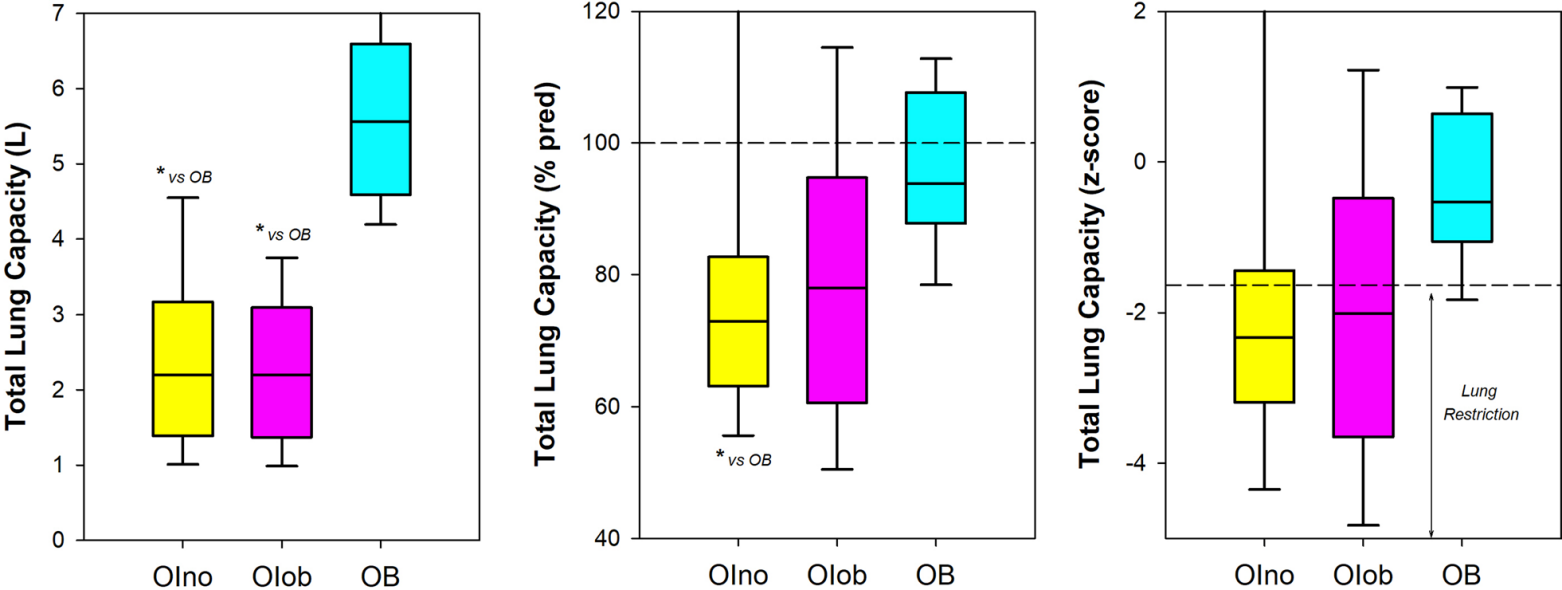
Box-plot representing the median (line within the box), the 10th (whisker below the box), 25th (boundary of the box closest to zero), the 75th (boundary of the box farthest from zero) and the 90th (whisker above the box) percentiles of the total vital capacity expressed as absolute value (left panel), as percentage of the predicted values (middle panel) and z-score (right panel) in patients without obesity affected by Osteogenesis Imperfecta (OIno), in patients with obesity affected by Osteogenesis Imperfecta (OIob) and in subjects with essential obesity not affected by Osteogenesis Imperfecta (OB). *: *p* < 0.05

**Fig. 3:**
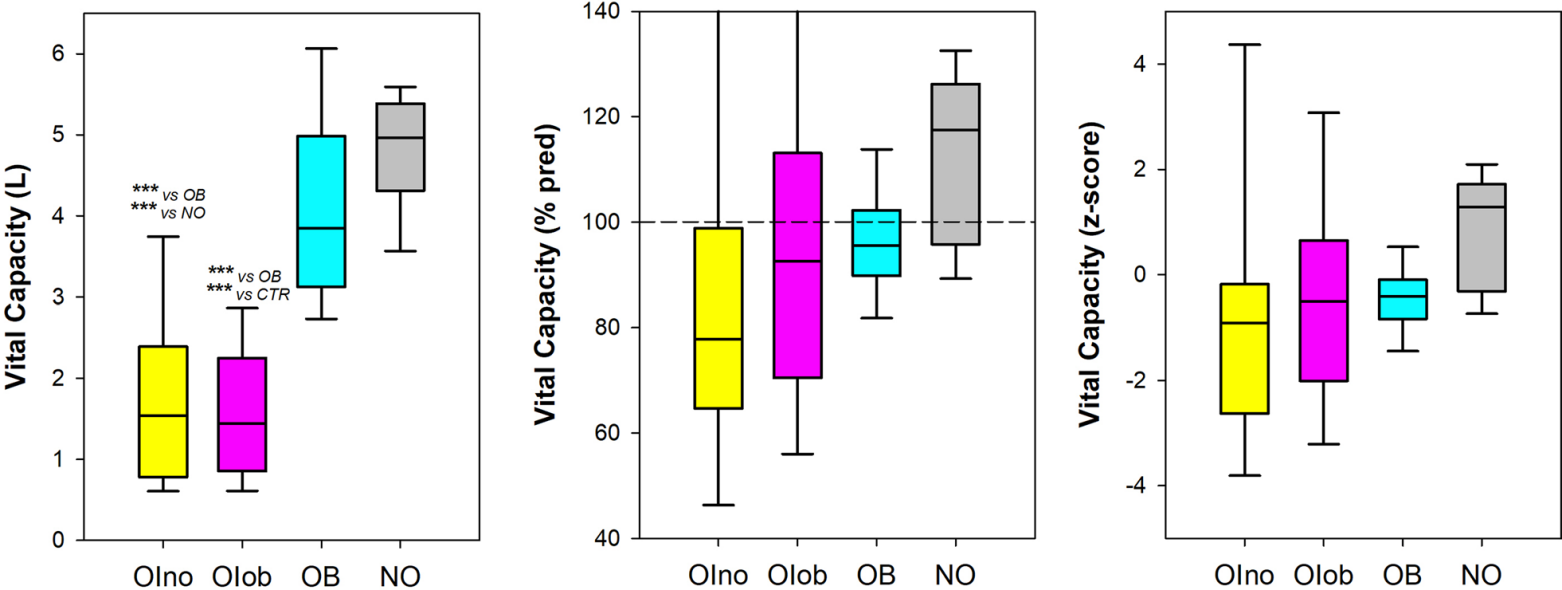
Box-plot representing the median (line within the box), the 10th (whisker below the box), 25th (boundary of the box closest to zero), the 75th (boundary of the box farthest from zero) and the 90th (whisker above the box) percentiles of the forced vital capacity expressed as absolute value (left panel), as percentage of the predicted values (middle panel) and z-score (right panel) in patients without obesity affected by Osteogenesis Imperfecta (OIno), in patients with obesity affected by Osteogenesis Imperfecta (OIob), in subjects with essential obesity not affected by Osteogenesis Imperfecta (OB) and in otherwise healthy without obesity subjects not affected by Osteogenesis Imperfecta (NO). ***: *p* < 0.001

Restricted lung pattern (i.e.: z-score of TLC < − 1.64) was diagnosed in 10 OIno (77%), 9 OIob (65%), and 1 OB (10%) subjects (Fig. 2). FVC was lower in both groups of patients affected by moderate or severe Osteogenesis Imperfecta only when expressed as an absolute value (Fig. 3 and Table 1 online supplement). OSAS was diagnosed in 4 OIno (30%) and 9 OIob (64%) according to AHI and ODI (Table 1 online supplement).

### Ventilatory and thoraco-abdominal pattern at rest

**Fig. 4:**
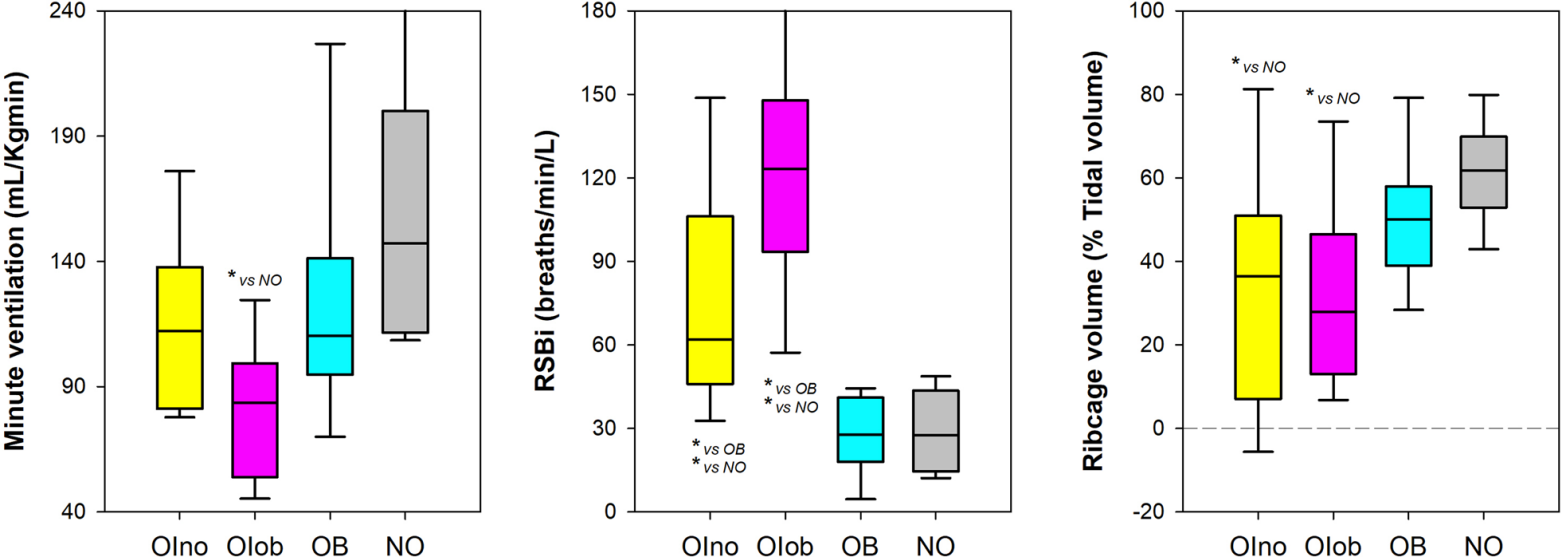
Box-plot representing the median (line within the box), the 10th (whisker below the box), 25th (boundary of the box closest to zero), the 75th (boundary of the box farthest from zero), and the 90th (whisker above the box) percentiles of minute ventilation (left panel), rapid and shallow breathing index (middle panel), and pulmonary ribcage percentage contribution to tidal volume (right panel) at rest in seated position in patients without obesity affected by Osteogenesis Imperfecta (OIno), in patients with obesity affected by Osteogenesis Imperfecta (OIob), in subjects with essential obesity not affected by Osteogenesis Imperfecta (OB) and in otherwise healthy without obesity subjects not affected by Osteogenesis Imperfecta (NO). *: *p* < 0.05

Seated position: patients with obesity affected by moderate or severe Osteogenesis Imperfecta breathed with reduced tidal volume and higher respiratory rate resulting in hypoventilation. Rapid and shallow breathing characterised both groups of subjects affected by moderate or severe Osteogenesis Imperfecta. The ribcage contribution was lower in both groups of patients affected by moderate or severe Osteogenesis Imperfecta and it was negative in 3 (23%) OIno and 3 (21%) OIob (Fig. 4, Table 1 and Table 1 online supplement).

**Fig. 5:**
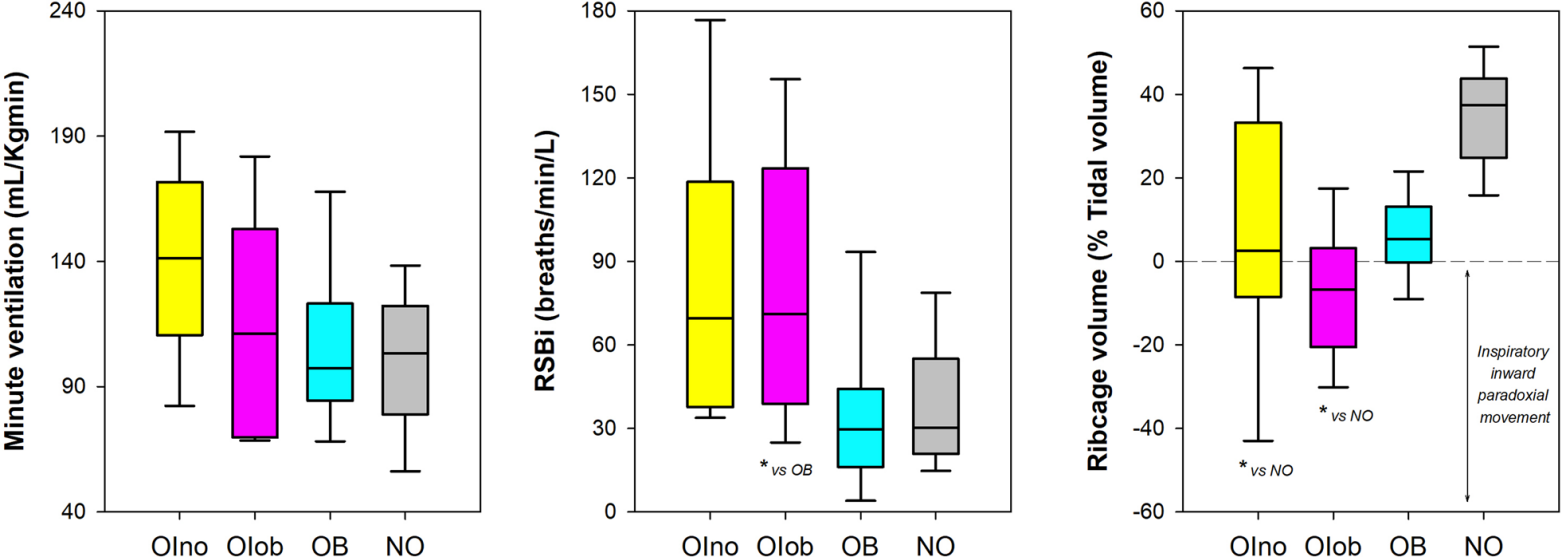
Box-plot representing the median (line within the box), the 10th (whisker below the box), 25th (boundary of the box closest to zero), the 75th (boundary of the box farthest from zero) and the 90th (whisker above the box) percentiles of minute ventilation (left panel), rapid and shallow breathing index (middle panel), and pulmonary ribcage percentage contribution to tidal volume (right panel) at rest in supine position i in patients without obesity affected by Osteogenesis Imperfecta (OIno), in patients with obesity affected by Osteogenesis Imperfecta (OIob), in subjects with essential obesity not affected by Osteogenesis Imperfecta (OB) and in otherwise healthy without obesity subjects not affected by Osteogenesis Imperfecta (NO). *: p < 0.05

Supine position: the ventilatory pattern was similar among the four groups, while differences occurred in the thoraco-abdominal compartmentalization. Both groups of patients affected by moderate or severe Osteogenesis Imperfecta were characterised by reduced ribcage contribution, which was negative in 6 (46%) OIno, 11 (78%) OIob, and 1 (10%) OB (Fig. 5, Table 1 and Table 1 online supplement).

## Discussion

In this pilot study, we aimed to separate the impact of obesity on sleep quality and lung and chest wall expansion in moderate or severe Osteogenesis Imperfecta. The preliminary results suggested that moderate or severe Osteogenesis Imperfecta per se implies: (1) a 30% incidence of obstructive sleep apnoea syndrome; (2) a restricted lung pattern; (3) restricted ribcage expansion, and (4) rapid and shallow breathing in the seated position. The additional impact of obesity on moderate or severe Osteogenesis Imperfecta seemed to determine: (1) a higher incidence of obstructive sleep apnoea syndrome; (2) hypoventilation in the seated position; and (3) a higher incidence of paradoxical breathing while lying supine.

Our previous study showed that the pathophysiology of moderate or severe Osteogenesis Imperfecta ensues a dangerous vicious circle among breathing, sleep, and nutritional status. That study included only subjects with Osteogenesis Imperfecta and we found a high incidence of obstructive sleep apnoea and restricted breathing. However, we could not distinguish if these results were due to the disease, obesity, or a combination of the two [15]. In this study, we have grouped subjects affected by moderate or severe Osteogenesis Imperfecta according to obesity and compared them to peers not affected by the disease, with and without obesity. Although these preliminary results need to be confirmed on a larger scale, they provide interesting insight.

Obesity is a chronic, complex disease that can impair health by increasing the risk of type II diabetes, heart disease, and certain cancers. Obesity can also affect bone health and is associated with poor sleep quantity and quality [26]. The major contributing factors to obesity include, among others, eating behaviour and physical inactivity. A linear correlation between obesity and OSAS exists due to the fat deposits in the upper respiratory tract that narrow the airway. In people with obesity, the muscle activity in the upper respiratory tract is reduced, leading to hypoxic and apneic episodes during sleep.

Obesity is also frequently associated with lung restriction. However, subjects with essential obesity might not be characterised by a systematic restrictive lung pattern [18]. Indeed, the lung capacities of our small population of subjects with obesity non affected by Osteogenesis Imperfecta were not restricted. By contrast, our data confirmed that moderate or severe Osteogenesis Imperfecta causes a restrictive pattern on both the lung and the chest wall independently of the BMI. Because collagen is also present in lung tissue, altered gas exchange was postulated in patients affected by Osteogenesis Imperfecta [33]. This intrinsic collagen deficit in the lung tissue was shown in some mouse models of Osteogenesis Imperfecta. Dimori and colleagues showed that the impact of type I collagen alterations on the morphology and function of murine lung positively correlates with the severity of the extracellular matrix deficiency. Future studies should aim to determine whether these observations in mice translate to patients with Osteogenesis Imperfecta. It would be important to dissect the relative contribution of intrinsic lung defects and extrinsic thoracic skeletal defects to impaired lung function. The extrinsic thoracic skeletal defects include fragility and deformation of the bones, ligaments, and articular structures. Additionally, the combination of structural modifications of the spine (kyphoscoliosis) and the ribcage (*pectus carinatum* and horizontal brittle ribs) severely reduces thoracic mobility [16]. In our pilot study, the incidence of lung restriction was high in both groups of patients affected by moderate or severe Osteogenesis Imperfecta. Similarly, thoracic expansion was systematically reduced in both positions in the presence of Osteogenesis Imperfecta, independently of BMI. Obesity further aggravated paradoxical inward thoracic breathing in moderate or severe Osteogenesis Imperfecta, occurring mainly in the supine position and potentially impacting sleep. In the seated position, obesity restricted the tidal volume of patients affected by moderate or severe Osteogenesis Imperfecta. The increased breathing rate did not compensate for this restriction. Therefore, obesity triggered hypoventilation and rapid and shallow breathing in the seated position in moderate or severe Osteogenesis Imperfecta. For all these reasons, it is crucial for patients affected by Osteogenesis Imperfecta to avoid obesity. The prevention and contrast of obesity is a societal problem, as obesity is one of the most blatantly visible public health problems [27]. Individuals with obesity are at risk of several medical conditions that can lead to further morbidity and mortality. Addressing the obesity epidemic is crucial for the general healthy population, but even more so in the presence of any disease. In this pilot study, moderate or severe Osteogenesis Imperfecta appears to be an aggravating factor that, in addition to obesity, contributes to causing OSAS and restricting lung and chest wall.

Antonaglia and Passuti found a frequency of approximately 20% of OSAS in normal-weight patients [28]. The frequency in our subjects without obesity affected by moderate or severe Osteogenesis Imperfecta was higher than 20% and comparable to the value found in a Danish population of Osteogenesis Imperfecta [20]. This indicates that intrinsic characteristics of moderate or severe Osteogenesis Imperfecta make patients prone to OSAS independently of body weight. These anatomical pathological prevalent traits include craniofacial conformations of reduced dimensions, laxity of the soft palate, and macroglossia, resulting is a more significant collapse of the upper airways in patients, even those without obesity [28].

OSAS prevalence can be around 40% in obese males with BMIs greater than 30 kg/m^2^ [29], while in severely obese patients (BMI > 40 kg/m^2^) the prevalence of OSAS ranges from 55 to 90% [30, 31]. The median BMI of our subjects with obesity affected by moderate or severe Osteogenesis Imperfecta was 35.7 40 kg/m^2^. The 64% frequency of OSAS that we found was slightly higher than in the obese population without Osteogenesis Imperfecta. The prevalence of OSAS in people with obesity undergoing bariatric surgery is 77% [32]. This percentage is not far from the value found in our subjects with obesity affected by Osteogenesis Imperfecta, although the BMI of our patients was lower than that of the bariatric patients (35 vs. 49.5) [30].

Interestingly, the majority of the OSAS diagnoses in our pilot study were new. Patients were unaware of the problem, although they frequently reported fatigue and tiredness. OSAS, therefore, risks being underdiagnosed in this population. Patients with Osteogenesis Imperfecta are high-risk individuals for OSAS for two reasons. Firstly, they exhibit congenital markers (cervical and mandible skeletal deformation) indicating predisposition. Secondly, the risk of developing obesity in Osteogenesis Imperfecta is above average or high (also in the pediatric population [34]), due to small body size, poor mobility, and lack of physical activity. In addition, the nutritional status and dietary requirements of patients with Osteogenesis Imperfecta differ from the normal population [19, 35]. Addressing obesity in these patients is crucial, as obesity puts additional stress on weak bones. Fat negatively affects bone physiology, while poor muscle mass and strength correlate with an increased risk of fractures [35–37].

The only way to control, prevent, and manage obesity is through behavioural-based dietary and physical activity interventions. Exercise and physical activity are critical topics for individuals with Osteogenesis Imperfecta. Despite the many benefits associated with sport and exercise, most people with disabilities are insufficiently active. Physical inactivity is a significant problem among people with physical disabilities, who are more likely to use their condition as a barrier to physical activity. Due to brittle bones, children with Osteogenesis Imperfecta are more likely to be raised in protective enviroments and are less likely to be physically active later in life. Additionally, most physiotherapists or personal trainers lack expertise in handling subjects with Osteogenesis Imperfecta. Alternative programs in physical education and sport are not always available for these patients, representing a barrier to physical activity for Osteogenesis Imperfecta.

Regarding dietary intake, our previous study showed that patients with Osteogenesis Imperfecta tend to follow an unhealthy diet, characterised by an excess of saturated fats and simple sugars and a reduction in fibre and micronutrients intake, such as calcium, iron, and vitamin D. Both aspects contribute to an increase in body weight in the short and long term, primarily through an increase in body fat and related comorbidities (e.g., impairments in glucose and lipid profiles). By contrast, a Mediterranean diet, rich in vegetable fats from olive oil, nuts and fatty fish, and fibres from whole grains and vegetables, was effective in improving body composition in terms of the ratio between lean and fat mass, as well as enhancing glucose and lipid profiles. These results suggest that a restricted Mediterranean diet, especially if combined with a structured and personalised physical activity, could improve body composition and overall nutritional status [19].

This study has several limitations. Although Osteogenesis Imperfecta is a rare disorder, the low number of subjects in each subgroup is a significant shortcoming. Due to the relatively small study populations, we excluded the mildest type of Osteogenesis Imperfecta because it is a non-deforming form, frequently not diagnosed until adulthood. Therefore, there was a risk of a negligible impact of the disease in considering the mildest form in this small study group. Future studies with a larger study group should include other forms of Osteogenesis Imperfecta [38], and consider other factors such as sex, age and lifestyle. This type of study requires recruiting closely matching control participants. In our pilot study, the groups were matched according to age, BMI (similar between the two groups without obesity and similar between the two groups with obesity) and body composition (similar between the two groups with obesity). The lack of complete acquisitions in all groups (i.e.: nocturnal evaluation on subjects not affected by Osteogenesis Imperfecta, TLC and body composition measurement on subjects without obesity not affected by Osteogenesis Imperfecta) is another limit. These two limitations were mainly due to budget reasons. Nevertheless, many studies deal with OSAS in subjects not affected by Osteogenesis Imperfecta with and without obesity [28–31]. The incidence of obesity taken from the literature is therefore reliable, and it is reasonable to apply it to our small population. Furthermore, there are already studies using opto-electronic plethysmography on people with obesity who are not affected by Osteogenesis Imperfecta [18, 39–43].

Another important deficiency was the lack of data on nutritional habits and physical activity.

Despite these limitations, this pilot study provides new insights into moderate or severe Osteogenesis Imperfecta by discerning the effect of the disease and obesity on sleep and respiratory restriction, therefore encouraging additional research. The clinical implications of this pilot study confirm the recommendation for sleep evaluation in populations affected by moderate or severe Osteogenesis Imperfecta [14], even in patients without obesity. Based on the experience matured in this pilot study, future studies should be multicenter to allow quicker recruitment of diverse populations. A uniform implementation methodology is also essential for the generalizability of the results. Facilities for lung volume, sleep, body composition and nutritional measurements are easily available in many hospitals and clinical or medical centres. However, the availability of opto-electronic plethysmography is limited and mostly restricted to universities and research centres. We believe that opto-electronic plethysmography deserves financial investments because the quantification of paradoxical breathing and the non-volitional nature of the measurement are important factors for the study.

To conclude, intrinsic characteristics of the disease make moderate or severe Osteogenesis Imperfecta subjects prone to obstructive sleep apnea syndrome, restricted lung, and rapid and shallow breathing. The additional impact of obesity further increased the incidence of obstructive sleep apnea syndrome, paradoxical breathing lying supine, and hypoventilation in the seated position. Reversing obesity in Osteogenesis Imperfecta is even more challenging as knowledge of the diet and physical activity suited for these patients is still scarce.

## Supplementary Information

Additional file 1.

## Data Availability

The data supporting this study's findings are available upon request from the corresponding author ALM. The data are not publicly available due to restrictions since they contain information that could compromise the privacy of research participants.

## Abbreviations

AHI: Apnea–hypopnea index
BMC: Bone mineral content
BMI: Body mass index
BW: Body weight
DEXA: Duel-energy X-ray absorptiometry
FFM: Fat-free mass
FM: Fat mass
FMI: Fat mass index
FVC: Forced vital capacity
LM: Lean body mass
NO: Subjects without obesity not affected by Osteogenesis Imperfecta
OB: Subjects with obesity not affected by Osteogenesis Imperfecta
OI: Osteogenesis Imperfecta
OIob: Subjects with obesity affected by Osteogenesis Imperfecta
OIno: Subjects without obesity affected by Osteogenesis Imperfecta
OSAS: Obstructive sleep apnea syndrome
TLC: Total lung capacity
