# Supplementary material for: The impact of obesity on sleep, pulmonary and chest wall restriction in Osteogenesis Imperfecta: a pilot study
Source: Orphanet J Rare Dis. 2024 Dec 20;19:479. doi: 10.1186/s13023-024-03489-z (PMC11662544; doi:10.1186/s13023-024-03489-z)
Supplement: Supplementary file 1 — Additional file 1. [file 13023_2024_3489_MOESM1_ESM.docx]

**Table 1OS. Forced vital capacity, total lung capacity, sleep data, and respiratory pattern in patients without obesity affected by Osteogenesis Imperfecta (OIno), in patients with obesity affected by Osteogenesis Imperfecta (OIob), in subjects with essential obesity not affected by Osteogenesis Imperfecta (OB) and in otherwise healthy subjects without obesity not affected by Osteogenesis Imperfecta (NO).**

FVC: forced vital capacity; TLC: total lung capacity; AHI: apnea-hypoapnea index; ODI: oxygen desaturation index; RSBi: Rapid and Shallow Breathing index; 25p: 25^th^ percentile; 75p: 75^th^ percentile;
